# Causal associations of gastroesophageal reflux disease with sepsis and sepsis mortality within 28 days: A bidirectional 2-sample Mendelian randomization study

**DOI:** 10.1097/MD.0000000000050553

**Published:** 2026-09-04

**Authors:** Yangyang Tong, Jie Liu

**Affiliations:** a Department of Blood Transfusion, Zigong First People’s Hospital, Zigong, Sichuan, China; b Department of Critical Care Medicine, The First Affiliated Hospital of Guangzhou Medical University, Guangzhou, Guangdong, China.

**Keywords:** gastroesophageal reflux disease, Mendelian randomization, mortality, sepsis

## Abstract

Previous studies have demonstrated a potential correlation between gastroesophageal reflux disease (GERD) and sepsis. However, the causal association between GERD and sepsis remains unclear. We performed a bidirectional 2-sample Mendelian randomization (MR) analysis to investigate the causal associations of GERD with sepsis and sepsis mortality within 28 days. The inverse variance weighted method was used as a major method for the MR study, supplemented with multiple approaches, including MR-Egger, weighted median, and weighted mode. Then, the MR-Egger intercept test, Mendelian randomized polymorphism residual and outlier (MR-PRESSO) test, Cochran’s *Q* test, and leave-one-out test were conducted to assess the reliability of the MR results. Forward MR analysis indicated that GERD significantly increased the risk of sepsis (odd ratio [OR] = 1.36; 95% confidence interval [CI]: 1.21–1.52, *P* = 1.44E−07) and 28-day sepsis mortality (OR = 1.44; 95% CI: 1.08–1.92, *P* = .014). Reverse MR analysis indicated that sepsis didn’t causally affect GERD, while 28-day sepsis mortality showed a suggestive positive association with GERD (OR = 1.03; 95% CI: 1.01–1.06, *P* = .015), with a modest but statistically significant effect size. In addition, the MR analyses were found to be robust based on sensitivity tests, indicating no presence of heterogeneity or horizontal pleiotropy. Our study indicated that genetically predicted GERD increases the risk of sepsis and 28-day sepsis mortality, while genetically predicted 28-day sepsis mortality may also increase the risk of GERD, presenting a novel approach to the preventive strategy for sepsis and therapeutic perspectives in patients with GERD. However, given the lenient SNP threshold and small effect size in reverse MR analysis, as well as the exclusive European study cohort, additional studies are required to validate our findings.

## 1. Background

Sepsis and gastroesophageal reflux disease (GERD) are prevalent clinical disorders that impose significant global health hazards and economic burdens. Sepsis is a critical medical condition characterized by an imbalanced immune response to infection, leading to impaired organ function.^[1,2]^ Aberrations in inflammation, coagulation, immune function, metabolic pathways, and oxygen delivery collectively play a role in the progression of organ dysfunction and mortality.^[3]^ Sepsis has been a significant contributor to global morbidity throughout the years.^[4]^

GERD, a prevalent disorder of the digestive system, is characterized by the persistent reflux of stomach contents into the esophagus or trachea. This condition is consistently associated with various complications such as chronic obstructive pulmonary disease, asthma, idiopathic pulmonary fibrosis, and so on.^[5]^ The prevalence of GERD is significantly elevated in Europe and North America, exhibiting a consistent upward trajectory over the years.^[6]^ Prior research has established a correlation between GERD and pulmonary infection, as well as its potential impact on autoimmune function through modulation of autonomic nervous system activity.^[7–9]^ Generally, severe infection and dysregulated immune response contribute to developing sepsis.^[10]^ Gastroesophageal reflux, a common gastrointestinal motility disorder, is frequently observed in septic patients admitted to the intensive care unit.^[11]^

However, retrospective studies could not explain the causal mechanism between GERD and sepsis, due to shared risk factors that may introduce confounding. Mendelian randomization (MR) is a statistical technique employed to estimate the causal effects of exposure factors on outcomes. This analytical approach eliminates confounding factors and enables the identification of causal relationships between exposure and diseases.^[12]^ In the present study, we systematically evaluated the causal associations of GERD with sepsis and 28-day mortality in sepsis within a bidirectional 2-sample MR analysis using genome-wide association study (GWAS) summary data. The discoveries could have important clinical implications in terms of disease management and the formulation of therapeutic approaches.

## 2. Materials and methods

### 2.1. Study design

A well-conducted MR study needs to satisfy 3 assumptions: First, the instrumental variables (IVs) have a strong association with the exposures being studied; Second, IVs are not influenced by any confounding factors; Third, IVs only impact the outcome through the exposures.^[13]^

### 2.2. Data sources

The GWAS data for GERD were obtained from the IEU database (id: ebi-a-GCST90000514). The summary data included 6,02,604 European ancestry participants (1,29,080 individuals with GERD and 6,02,604 without) with a dataset of 23,20,781 single nucleotide polymorphisms (SNPs). The GWAS data for sepsis (11,643/4,74,841) and sepsis mortality within 28 days (1896/4,84,588) were also acquired from the IEU database (ieu-b-4980, ieu-b-5086). The information regarding the GWAS summary statistics can be found in Table 1. The study populations and cohorts consisted solely of individuals from European backgrounds, limiting generalizability to other racial groups. Formal power calculation was not performed because the analysis used summary-level data from published GWAS. However, given the large sample sizes of the included datasets, the study was adequately powered to detect modest causal associations.

**Table 1:** Details of the genome-wide association study summary data.

Phenotype	Participants (cases/controls)	Number of SNP	Ancestry	GWAS ID
GERD	6,02,484 (1,29,080/4,73,524)	23,20,781	European	ebi-a-GCST90000514
Sepsis	4,86,484(11,643/4,74,841)	1,22,43,539	European	ieu-b-4980
Sepsis (28-day death)	4,86,484 (1896/4,84,588)	1,22,43,487	European	ieu-b-5086

GERD = gastroesophageal reflux disease, GWAS = genome-wide association study, SNP = single nucleotide polymorphism.

### 2.3. Selection of IVs

For forward MR analysis, *P* < 5e^−8^ was deemed statistically significant for the inclusion of IVs in this study. In reverse MR analysis, a more lenient threshold of *P* < 5e^−6^ was used to screen for IVs associated with sepsis and sepsis mortality within 28 days to account for the limited number of available IVs.^[14]^ IVs needed to meet an linkage disequilibrium threshold of *r*^2^ < 0.001 within 10,000 kb, in order to prevent linkage imbalance.^[15]^ SNPs containing palindromic sequences were excluded. The effectiveness of each instrument was assessed by calculating the *F*-statistic through the equation: (*F* = β^2^/SE^2^), where BETA denotes the estimated genetic impact on exposure and SE represents the standard error associated with this genetic effect. An *F*-statistic > 10 was deemed to indicate a sufficiently robust instrument with minimal potential for bias.^[16,17]^ Missing SNPs in the outcome were discarded. To address the gene-environment equivalence assumption, we used genetic instruments robustly associated with GERD that are largely unconfounded by lifestyle factors. The Phenoscanner V2 (http://www.phenoscanner.medschl.cam.ac.uk/) was applied to detect potential confounders (smoking, alcoholic intake, body height, body mass index, or diabetes^[18-23]^), and any SNPs linked to these confounders were removed before the analysis.^[24]^ Sensitivity analyses were conducted to evaluate potential violations of this assumption.

### 2.4. MR analysis and sensitivity analyses

The analysis was performed by employing the “TwoSampleMR” package in R software (version 4.4.0). The research protocols and details have not been preregistered. Four distinct methods, including inverse variance weighted (IVW), weighted median, weighted mode, and Mendelian randomization-Egger (MR-Egger) regression, were employed within this R package. The IVW method served as the primary analytical approach in this study.^[25,26]^ The fixed effects model is typically preferred when there is no heterogeneity (*P*-value exceeding .05), while the random effects model is favored in the presence of heterogeneity (*P*-value below .05).^[27]^ The *Q* test was used to detect any possible deviations from the assumption of heterogeneity in the association between individual IVs, and a *Q*-derived *P* < .05 will be considered the existence of heterogeneity.^[28]^ Besides, we investigated the presence of horizontal pleiotropy by employing MR-Egger regression and the Mendelian Randomized Polymorphism Residual and Outlier Global test. A significant threshold of *P* < .05 will indicate the existence of horizontal pleiotropy, implying that IVs may affect outcomes through other pathways, leading to false positives.^[29]^ The leave-one-out method was used to assess the influence of single SNPs on the outcome.^[30]^

### 2.5. Ethical approval

No ethics approval was required as the data utilized in this research were obtained from publicly available datasets.

## 3. Results

### 3.1. Results of forward MR analysis

**Figure 1.**
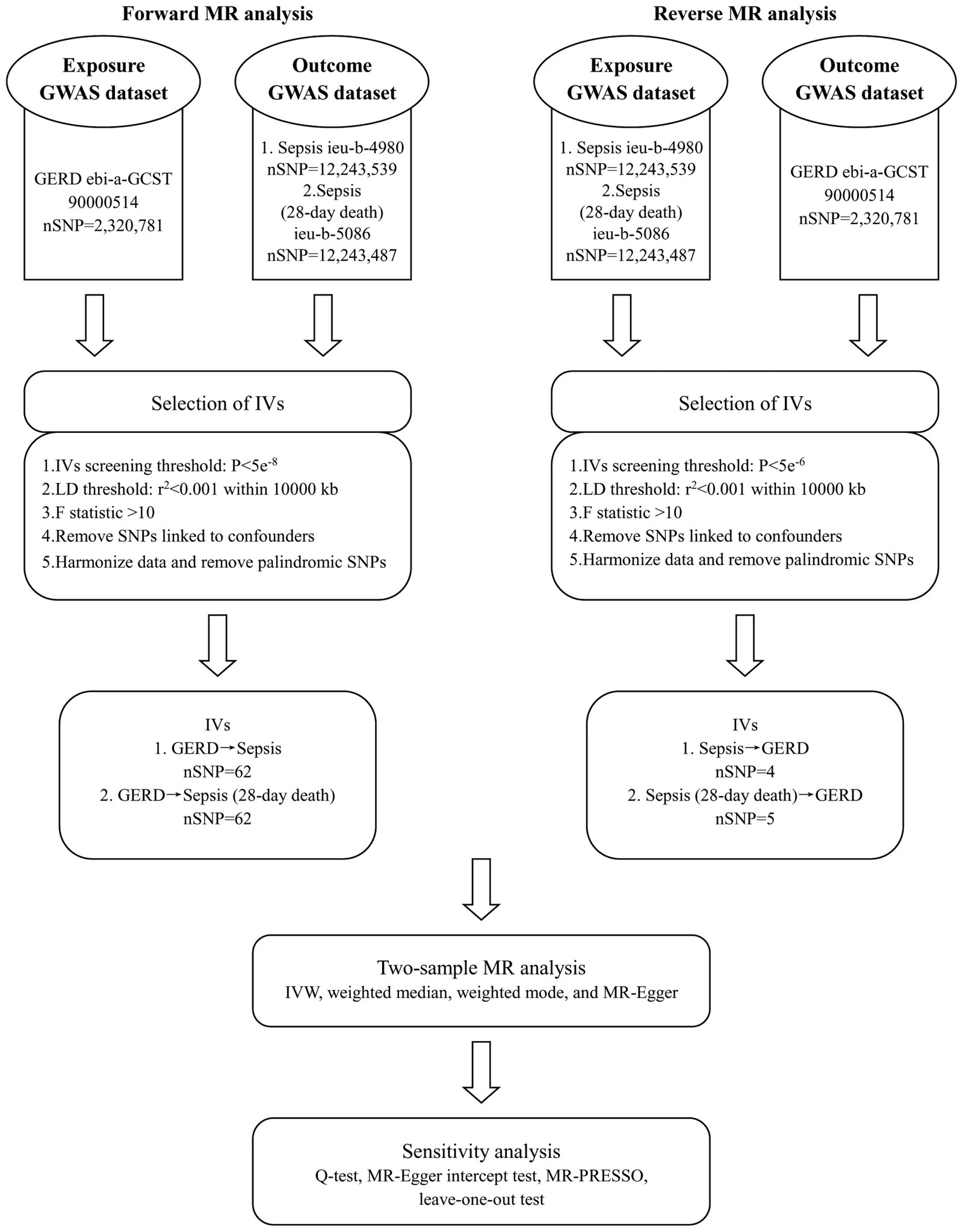
MR flow diagram. A bidirectional 2-sample MR analysis was used to examine the causal association between GERD and sepsis/sepsis (28-day death). Genetic instrumental variables were rigorously screened with a threshold of *P* < 5e^−8^ in forward MR analysis (*P* < 5e^−6^ in reverse MR analysis), LD clumping (*r*^2^ < 0.001, 10,000 kb), *F*-statistic > 10, confounder-related SNP exclusion, and palindromic SNP removal after data harmonization. Valid instrumental variables were used for 2-sample MR analysis with Inverse variance weighted, weighted median, weighted mode, and MR-Egger methods. Cochran’s *Q* test, MR-Egger intercept test, MR-PRESSO and leave-one-out sensitivity analyses were adopted to evaluate the reliability of causal results. GERD = gastroesophageal reflux disease, GWAS = genome-wide association, IV = instrumental variables, IVW = inverse variance weighted, LD = linkage disequilibrium, MR = Mendelian randomization, MR-PRESSO = Mendelian Randomized Polymorphism Residual and Outlier, nSNP = number of single nucleotide polymorphisms, SNP = single nucleotide polymorphism.

**Figure 2.**
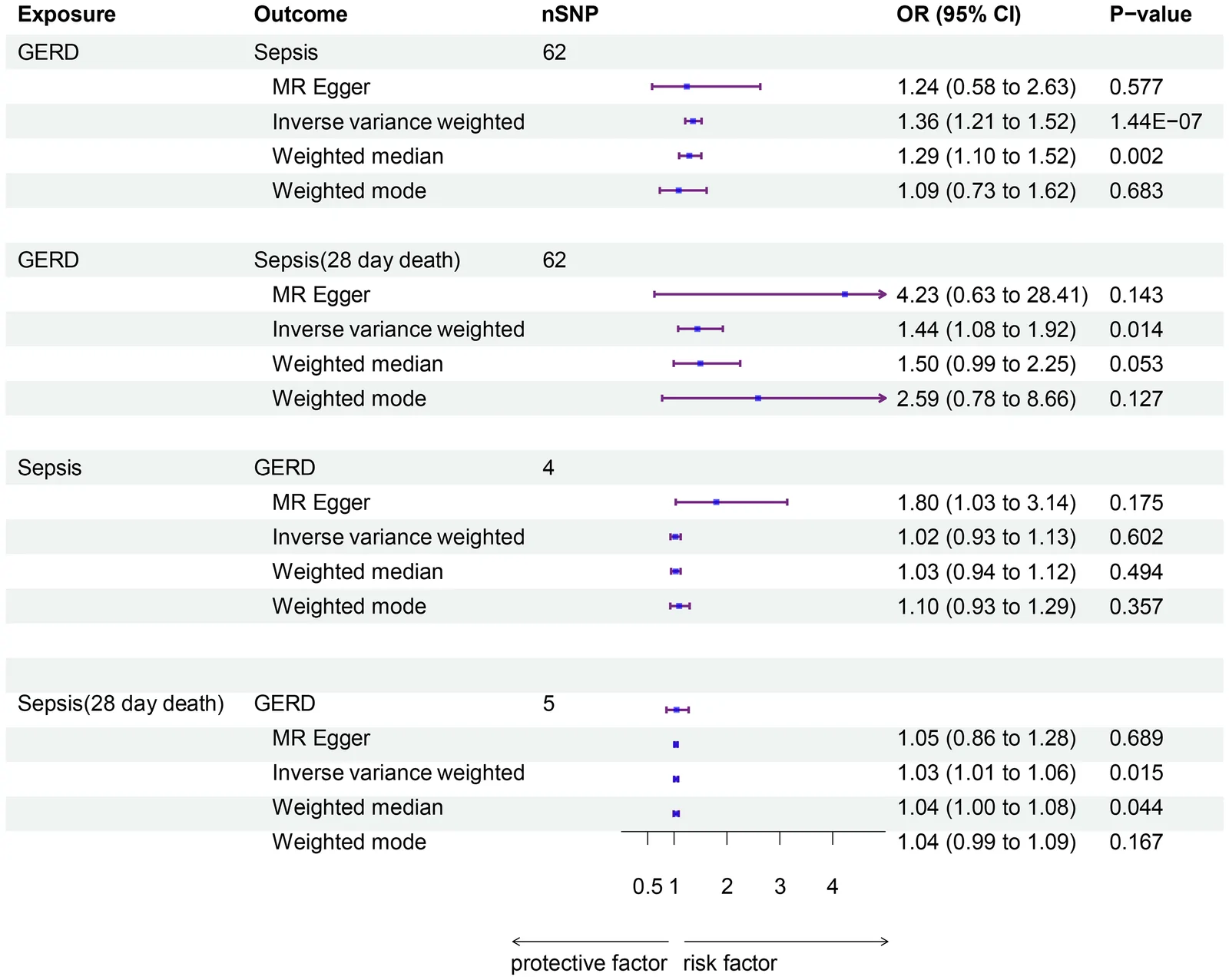
Forest plot for MR results. Results are shown for the different methods of MR analysis used in this study: MR-Egger, inverse variance weighted, weighted median, and weighted mode. This denotes the causal effect size for GERD on sepsis/sepsis (28-day death) and sepsis/sepsis (28-day death) on GERD. CI = confidence interval, GERD = gastroesophageal reflux disease, MR = Mendelian randomization, nSNP = number of single nucleotide polymorphisms, OR = odds ratio.

**Figure 3.**
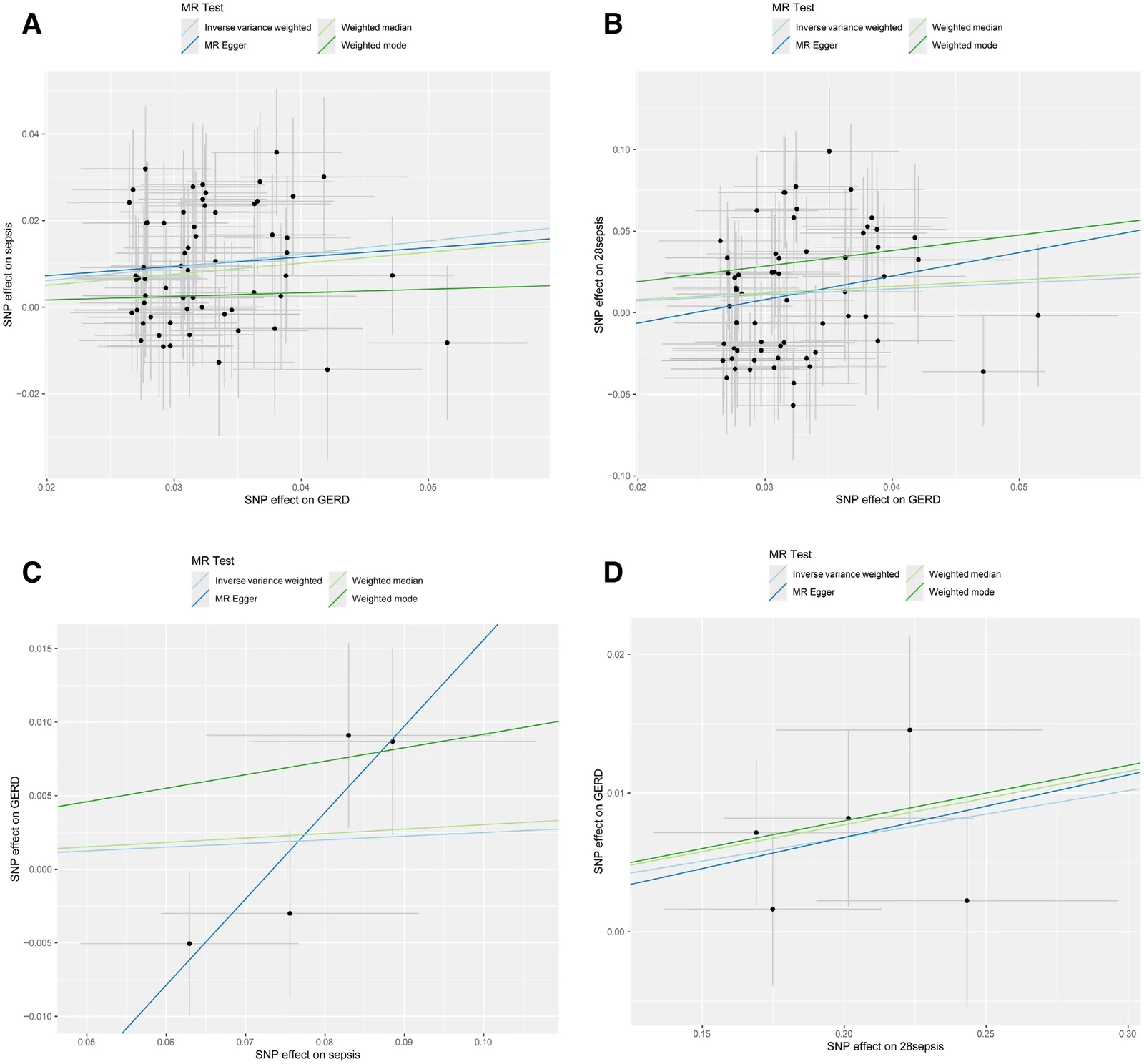
Scatter plot of SNPs. Visually estimate the causal effect for each individual SNP regarding GERD on (A) sepsis and (B) sepsis mortality within 28 days; Visually estimate the causal effect for each individual SNP regarding (C) sepsis and (D) sepsis mortality within 28 days on GERD. GERD = gastroesophageal reflux disease, MR = Mendelian randomization, SNP = single nucleotide polymorphism.

The SNPs from GWAS data were screened according to the steps outlined in the methods section. We initially screened 80 SNPs for GERD. The confounding SNPs were subsequently queried using Phenoscanner V2 and eliminated, among which are *rs17379561, rs13107325, rs329122, rs9373363, rs215614, rs4382592, rs2734839, rs9529055, rs12967855, rs2838771, rs7612999, rs903959, rs9517313.* Then we extracted IVs from the GWAS data of sepsis, and 65 SNPs were included. Additionally, 3 SNPs were excluded for being palindromic with intermediate allele frequencies, including *rs2145318, rs2358016,* and *rs957345.* Finally, a total of 62 SNPs were selected for the MR analysis. In the same way, 62 SNPs for sepsis mortality within 28 days were included in the MR analysis. The detailed procedure for SNP selection is shown in Figure 1. The detailed data of IVs and their F-values can be found in Table S1, Supplemental Digital Content 1 and Table S2, Supplemental Digital Content 2. As seen in Figure 2, the IVW analysis revealed a heightened susceptibility to sepsis among patients with GERD (odd ratio [OR] = 1.36; 95% confidence interval [CI]: 1.21–1.52, *P* = 1.44E−07). The estimates of the other 3 methods aligned with IVW. In addition, a similar causal correlation can be observed between GERD and sepsis mortality within 28 days (OR = 1.44; 95% CI: 1.08–1.92, *P* = .014), while the other 3 methods showed a consistent direction with IVW. The results of the forward MR analysis can be found in Table S5, Supplemental Digital Content 3. The scatter plots illustrating the corresponding associations are presented in Figure 3.

**Figure 4.**
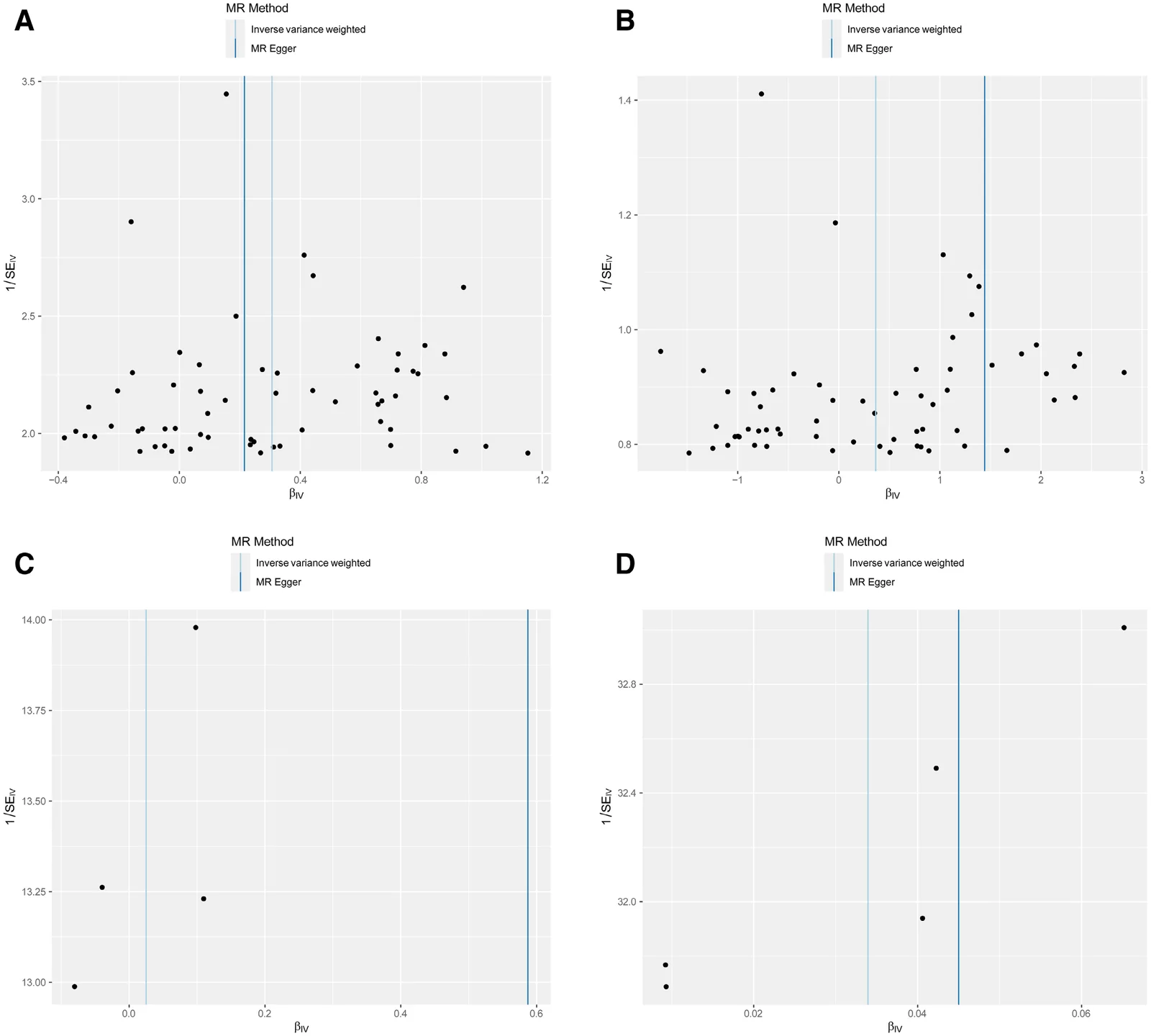
Funnel plot. The arrangement of dots in each funnel plot displayed a symmetrical pattern, indicating no significant publication bias. (A) Funnel plot of genetically predicted GERD on sepsis; (B) Funnel plot of genetically predicted GERD on sepsis mortality within 28 days; (C) Funnel plot of genetically predicted sepsis on GERD; (D) Funnel plot of genetically predicted sepsis mortality within 28 days on GERD. Abbreviations: GERD = gastroesophageal reflux disease, MR = Mendelian randomization, SE = standard error.

The robustness of forward MR results was demonstrated through a range of sensitivity tests. The MR-Egger intercept test and the Mendelian Randomized Polymorphism Residual and Outlier global test indicated the absence of horizontal pleiotropy on forward MR analysis (all *P* > .05; Table 2), and Cochran’s *Q* test showed no heterogeneity among the IVs (*P* > .05; Table 3). The distribution of dots in the funnel plot exhibited balanced symmetry (Fig. 4). The MR results were not significantly influenced by any SNPs, as indicated by the leave-one-out sensitivity analysis (Fig. S1, Supplemental Digital Content 4 and Fig. S2, Supplemental Digital Content 5).

**Table 2:** The results of horizontal pleiotropy.

Exposure	Outcome	MR-Egger intercept test			MR-PRESSO global test	
		Intercept	SE	*P*	RSS obs	*P*
GERD	Sepsis	0.003	0.012	.811	45.973	.946
GERD	Sepsis (28-day death)	−0.035	0.031	.267	68.349	.306
Sepsis	GERD	−0.043	0.022	.184	8.685	.267
Sepsis (28-day death)	GERD	−0.002	0.020	.920	3.778	.700

GERD = gastroesophageal reflux disease, MR = Mendelian randomization, RSS = residual sum of square, SE = standard error.

**Table 3:** The results of Cochran’s *Q* test.

Exposure	Outcome	Method	*Q*	*Q*_df	*Q*_pval
GERD	Sepsis	MR-Egger	44.425	60	0.934
		Inverse variance weighted	44.483	61	0.945
GERD	Sepsis (28-day death)	MR-Egger	64.631	60	0.318
		Inverse variance weighted	65.985	61	0.309
Sepsis	GERD	MR-Egger	0.925	2	0.630
		Inverse variance weighted	4.911	3	0.178
Sepsis (28-day death)	GERD	MR-Egger	2.394	3	0.495
		Inverse variance weighted	2.406	4	0.662

GERD = gastroesophageal reflux disease, MR = Mendelian randomization.

### 3.2. Results of reverse MR analysis

We conducted a reverse MR analysis to evaluate whether sepsis or sepsis mortality within 28 days causally affects GERD. First, we screened 14 SNPs for sepsis and 16 SNPs for sepsis mortality within 28 days. Then we extracted IVs from the GWAS data of GERD. Then 4 SNPs for sepsis and 5 SNPs for sepsis mortality within 28 days were included. The detailed procedure for SNP selection is shown in Figure 1. The detailed data of IVs and their *F*-values can be found in Table S3, Supplemental Digital Content 6 and Table S4, Supplemental Digital Content 7. The associations of these SNPs with confounding factors were not identified through Phenoscanner V2. As shown in Figure 2, sepsis did not exhibit a significant effect on GERD (OR = 1.02; 95% CI: 0.93–1.13, *P* = .602) in the IVW model, whereas 28-day sepsis mortality showed a suggestive positive association with GERD (OR = 1.03; 95% CI: 1.01–1.06, *P* = .015), with a modest but statistically significant effect size. The results of the reverse MR analysis can be found in Table S5, Supplemental Digital Content 3. The scatter plots illustrating the corresponding associations are presented in Figure 3.

As shown in Table 3, the Cochran *Q* statistic test didn’t reveal significant heterogeneity between instrument SNP effects (*P* > .05), and the results of directed pleiotropy shared no significant findings (all *P* > .05; Table 2). The arrangement of dots in the funnel plot displayed a symmetrical balance (Fig. 4). The leave-one-out sensitivity analysis revealed that no SNPs significantly affected the results (Fig. S3, Supplemental Digital Content 8 and Fig. S4, Supplemental Digital Content 9).

## 4. Discussion

In the present study, we conducted a bidirectional 2-sample MR analysis using publicly available GWAS summary statistics to investigate the causal associations of GERD with sepsis and 28-day mortality. The results demonstrated that GERD was associated with the risk of sepsis and 28-day sepsis mortality, while sepsis was not found to be causally associated with an increased susceptibility to GERD. Additionally, our study indicated that genetically predicted 28-day sepsis mortality may also increase the risk of GERD, with a modest but statistically significant effect size. Extensive sensitivity analyses showed that these associations were robust and showed consistent findings.

GERD and sepsis are 2 prevalent clinical disorders that impose significant global health hazards and economic burdens. However, there is limited literature exploring the causal association between them. In forward MR analysis, we found that GERD was a risk factor for sepsis and sepsis mortality within 28 days. The observed OR of 1.36 for sepsis and 1.44 for 28-day mortality suggests moderate increases in risk related to genetically predicted GERD. The presence of GERD may heighten the susceptibility to sepsis and increase sepsis mortality within 28 days by inducing recurrent respiratory infections and impairing immune system functionality. Previous studies have indicated a positive association between GERD and an elevated susceptibility to pneumonia.^[7,31]^ GERD may lead to the dissemination of Escherichia coli from the gastrointestinal tract to the respiratory tract, thereby resulting in infectious diseases.^[31]^ In addition, the presence of GERD is often accompanied by aberrant motility of the lower esophageal sphincter and vagal nerve dysfunction.^[8]^ The malfunctioning of the vagal nerve system leads to a disruption in the cholinergic anti-inflammatory pathway, leading to an imbalance in the immune system, increased levels of inflammatory substances in the bloodstream, and an intensified immune reaction.^[9]^ Furthermore, a population-based study unveiled the prevalence of insomnia among patients with GERD.^[32]^ Another prospective population-based study found that patients with insomnia have decreased immune function and an increased risk of bloodstream infection.^[33]^ Chronically elevated plasma levels of inflammatory factors in patients with GERD may also contribute to an increased susceptibility to sepsis and higher 28-day mortality rates.

Gastrointestinal motility disorders commonly present in the intensive care unit, serve as reliable indicators of heightened mortality rates and prolonged stays in this critical setting, and sepsis emerges as one of the most hazardous factors contributing to gastrointestinal motility disorders.^[11]^ Relevant evidence from previous studies suggested that sepsis might elevate the susceptibility to gastroesophageal reflux. In reverse MR analysis, our findings indicated that sepsis didn’t confer an elevated risk for GERD. However, our findings suggested that sepsis mortality within 28 days might be a risk factor for GERD. The OR of 1.03 indicates a slight increase in the risk of GERD associated with sepsis mortality within 28 days. The finding indicated that septic patients with a higher mortality rate may be more susceptible to developing GERD. In sepsis patients with high mortality rates, sympathetic nerve abnormalities are more prominent, leading to slower gastrointestinal peristalsis.^[34,35]^ In addition, they require more continuous mechanical ventilation, which may lead to a persistent rise in intra-abdominal pressure and increase the risk of GERD. Mechanistically, this finding seems explicable. The large sample size of the GWAS datasets provided high statistical power to detect this small but genuine signal, which might be undetectable in smaller observational studies. MR studies possess an inherent advantage over observational studies by effectively controlling for confounding factors, thereby elucidating a causal relationship between exposure and outcome. However, given the relatively small effect size, the finding should still be interpreted with caution. The elucidation of the precise mechanisms necessitates further inquiries.

The present study employed a bidirectional 2-sample MR to investigate the causal relationship between GERD and sepsis, as well as its impact on 28-day mortality in sepsis. This study represented the first application of genetic instruments derived from publicly available GWAS data to establish causal associations between GERD and sepsis, as well as sepsis mortality within 28 days. A variety of sensitivity tests were conducted to enhance the reliability of the conclusions. The current study also has several limitations. First, the present analysis was conducted on European ancestry, limiting generalizability to other racial groups. Notably, sepsis represents a major global health threat with a particularly high burden in low- and middle-income countries, where the incidence, mortality, and long-term complications of sepsis differ considerably from those in European populations. Future studies extending this MR framework to diverse ancestral populations are warranted to evaluate the consistency of causal associations across different genetic architectures, lifestyles, and healthcare systems, which will enhance the global applicability of our findings, especially for regions most affected by sepsis. Second, in the reverse MR analysis, a more lenient *P*-value threshold was used to include more IVs and improve statistical power. *F*-statistics were calculated for all variants, with all values > 10, confirming adequate instrument strength. Nevertheless, the relaxed threshold may increase the chance of including weaker instruments, and the observed effect size was relatively small, so reverse MR results should be interpreted with caution. Third, due to data constraints, subgroup analyses for gender and age were not performed. Fourth, despite conducting thorough sensitivity analyses, it proved challenging to completely eliminate the presence of horizontal pleiotropy.

## 5. Conclusions

In summary, our study indicated that genetically predicted GERD increases the risk of sepsis and 28-day sepsis mortality, while genetically predicted 28-day sepsis mortality may also increase the risk of GERD, presenting a novel approach to the preventive strategy for sepsis and therapeutic perspectives in patients with GERD. However, given the lenient SNP threshold and small effect size in reverse MR analysis, as well as the exclusive European study cohort, additional studies are required to validate our findings.

## Author contributions

**Conceptualization:** Yangyang Tong, Jie Liu.

**Data curation:** Yangyang Tong, Jie Liu.

**Formal analysis:** Yangyang Tong, Jie Liu.

**Funding acquisition:** Jie Liu.

**Investigation:** Yangyang Tong, Jie Liu.

**Methodology:** Jie Liu.

**Project administration:** Jie Liu.

**Resources:** Jie Liu.

**Software:** Yangyang Tong, Jie Liu.

**Supervision:** Jie Liu.

**Validation:** Jie Liu.

**Visualization:** Jie Liu.

**Writing – original draft:** Yangyang Tong, Jie Liu.

**Writing – review & editing:** Jie Liu.


















