# Supplementary material for: Causal associations of gastroesophageal reflux disease with sepsis and sepsis mortality within 28 days: A bidirectional 2-sample Mendelian randomization study
Source: Medicine (Baltimore). 2026 Sep 4;105(36):e50553. doi: 10.1097/MD.0000000000050553 (PMC13549649; doi:10.1097/MD.0000000000050553)
Supplement: Supplementary file 1 [file medi-105-e50553-s001.docx]

Table S1. Genetic data for the MR analysis on the effect of GERD on sepsis.

| SNP | chr.exposure | pos.exposure | effect allele | reference allele | exposure | | |
| --- | --- | --- | --- | --- | --- | --- | --- |
|  |  |  |  |  | beta | se | p |
| rs10010963 | 4 | 2E+08 | T | C | -0.027 | 0.005 | 4.92E-08 |
| rs1011407 | 2 | 6E+07 | G | A | -0.042 | 0.007 | 1.09E-08 |
| rs10133111 | 14 | 1E+08 | A | G | 0.042 | 0.007 | 1.35E-10 |
| rs1021363 | 10 | 1E+08 | G | A | -0.031 | 0.005 | 5.10E-10 |
| rs10837002 | 11 | 4E+07 | G | C | 0.028 | 0.005 | 4.03E-08 |
| rs11762636 | 7 | 2E+06 | A | C | -0.051 | 0.006 | 1.88E-16 |
| rs11953061 | 5 | 1E+08 | T | C | 0.028 | 0.005 | 3.10E-08 |
| rs12204714 | 6 | 2E+08 | T | C | -0.029 | 0.005 | 7.92E-09 |
| rs12357321 | 10 | 2E+07 | A | G | 0.032 | 0.005 | 1.33E-09 |
| rs12453010 | 17 | 5E+07 | T | C | 0.030 | 0.005 | 1.75E-09 |
| rs12598916 | 16 | 6E+07 | G | C | -0.033 | 0.005 | 6.87E-10 |
| rs12997558 | 2 | 4E+07 | A | G | 0.028 | 0.005 | 3.04E-08 |
| rs1334297 | 13 | 6E+07 | A | G | -0.039 | 0.005 | 1.14E-12 |
| rs13409451 | 2 | 1E+08 | G | A | -0.028 | 0.005 | 1.93E-08 |
| rs1431196 | 18 | 5E+07 | G | A | 0.032 | 0.005 | 2.65E-11 |
| rs1479405 | 12 | 2E+07 | T | C | 0.031 | 0.005 | 9.85E-10 |
| rs1510719 | 4 | 1E+08 | C | T | -0.039 | 0.005 | 3.84E-15 |
| rs1592757 | 5 | 1E+08 | C | G | 0.031 | 0.005 | 6.00E-10 |
| rs1596747 | 2 | 2E+08 | G | A | 0.031 | 0.005 | 1.00E-10 |
| rs1716171 | 12 | 1E+08 | T | C | 0.038 | 0.006 | 7.82E-11 |
| rs1883842 | 20 | 4E+07 | G | T | 0.031 | 0.005 | 9.27E-09 |
| rs1937450 | 1 | 7E+07 | G | T | 0.032 | 0.005 | 7.07E-11 |
| rs2016933 | 3 | 7E+07 | G | C | -0.031 | 0.005 | 1.04E-08 |
| rs2023878 | 19 | 2E+07 | T | C | -0.036 | 0.006 | 3.03E-09 |
| rs2043539 | 7 | 1E+07 | A | G | 0.027 | 0.005 | 2.24E-08 |
| rs2106353 | 7 | 1E+08 | T | G | 0.037 | 0.006 | 1.37E-10 |
| rs2164300 | 4 | 7E+07 | T | C | -0.026 | 0.005 | 4.13E-08 |
| rs2240326 | 3 | 5E+07 | A | G | -0.047 | 0.005 | 1.13E-22 |
| rs2396133 | 7 | 1E+08 | G | A | 0.029 | 0.005 | 1.11E-09 |
| rs2396766 | 7 | 1E+08 | A | G | 0.032 | 0.005 | 2.34E-11 |
| rs2744961 | 6 | 3E+07 | T | C | 0.029 | 0.005 | 5.81E-09 |
| rs2782641 | 1 | 4E+07 | A | G | 0.027 | 0.005 | 4.33E-08 |
| rs2815749 | 1 | 7E+07 | G | A | 0.039 | 0.006 | 1.07E-10 |
| rs2834005 | 21 | 3E+07 | C | T | 0.030 | 0.005 | 9.42E-09 |
| rs324769 | 12 | 8E+07 | T | C | -0.027 | 0.005 | 3.05E-08 |
| rs3766823 | 1 | 3E+07 | A | G | 0.039 | 0.006 | 7.09E-10 |
| rs3793577 | 9 | 2E+07 | G | A | 0.027 | 0.005 | 2.49E-08 |
| rs3863241 | 8 | 7E+07 | T | C | 0.032 | 0.005 | 1.49E-11 |
| rs4300861 | 2 | 2E+07 | T | C | 0.031 | 0.005 | 5.43E-10 |
| rs4713692 | 6 | 3E+07 | T | C | -0.028 | 0.005 | 3.06E-08 |
| rs569356 | 1 | 3E+07 | G | A | -0.038 | 0.007 | 4.07E-08 |
| rs6711584 | 2 | 1E+08 | A | G | 0.032 | 0.005 | 2.66E-11 |
| rs6722661 | 2 | 1E+08 | A | G | -0.032 | 0.005 | 1.15E-10 |
| rs6780459 | 3 | 1E+08 | T | A | 0.031 | 0.006 | 3.14E-08 |
| rs7032155 | 9 | 1E+08 | A | C | 0.028 | 0.005 | 1.63E-08 |
| rs7206608 | 16 | 8E+07 | G | C | 0.029 | 0.005 | 1.46E-08 |
| rs7241572 | 18 | 8E+07 | A | G | 0.037 | 0.006 | 9.49E-10 |
| rs7527682 | 1 | 2E+08 | G | A | -0.027 | 0.005 | 3.13E-08 |
| rs7541875 | 1 | 2E+08 | G | A | 0.027 | 0.005 | 1.61E-08 |
| rs761777 | 10 | 1E+08 | G | A | 0.035 | 0.006 | 4.71E-10 |
| rs7675588 | 4 | 8E+07 | A | C | -0.034 | 0.006 | 1.80E-08 |
| rs7685686 | 4 | 3E+06 | G | A | -0.028 | 0.005 | 1.14E-08 |
| rs773109 | 12 | 6E+07 | A | G | -0.038 | 0.005 | 8.71E-14 |
| rs7942368 | 11 | 8E+07 | T | C | -0.034 | 0.006 | 9.54E-09 |
| rs903678 | 1 | 2E+08 | A | G | 0.028 | 0.005 | 4.89E-08 |
| rs9372625 | 6 | 1E+08 | A | G | -0.038 | 0.005 | 2.62E-14 |
| rs9396740 | 6 | 2E+07 | A | G | -0.031 | 0.006 | 1.47E-08 |
| rs942065 | 14 | 9E+07 | A | G | 0.031 | 0.005 | 8.45E-10 |
| rs9542729 | 13 | 3E+07 | G | C | -0.036 | 0.006 | 1.41E-09 |
| rs9615905 | 22 | 5E+07 | T | C | 0.028 | 0.005 | 1.21E-08 |
| rs9636202 | 19 | 2E+07 | A | G | -0.035 | 0.005 | 1.51E-10 |
| rs9940128 | 16 | 5E+07 | A | G | 0.033 | 0.005 | 8.06E-12 |

| outcome | | | F statistics |
| --- | --- | --- | --- |
| beta | se | p |  |
| -0.007 | 0.014 | 0.606 | 29.7 |
| 0.014 | 0.021 | 0.491 | 32.7 |
| 0.030 | 0.018 | 0.102 | 41.2 |
| 0.006 | 0.014 | 0.656 | 38.6 |
| 0.001 | 0.014 | 0.944 | 30.1 |
| 0.008 | 0.018 | 0.644 | 67.7 |
| -0.002 | 0.014 | 0.876 | 30.6 |
| 0.006 | 0.014 | 0.647 | 33.3 |
| 0.016 | 0.015 | 0.271 | 36.8 |
| -0.009 | 0.014 | 0.527 | 36.2 |
| -0.011 | 0.015 | 0.488 | 38.1 |
| 0.019 | 0.014 | 0.173 | 30.7 |
| -0.007 | 0.016 | 0.639 | 50.6 |
| -0.007 | 0.014 | 0.640 | 31.6 |
| 0.023 | 0.014 | 0.090 | 44.4 |
| 0.028 | 0.015 | 0.057 | 37.4 |
| -0.016 | 0.014 | 0.255 | 61.8 |
| 0.014 | 0.014 | 0.336 | 38.3 |
| 0.009 | 0.014 | 0.533 | 41.8 |
| 0.003 | 0.017 | 0.880 | 42.3 |
| 0.013 | 0.015 | 0.413 | 33.0 |
| 0.019 | 0.014 | 0.178 | 42.5 |
| 0.000 | 0.015 | 0.978 | 32.8 |
| -0.003 | 0.017 | 0.845 | 35.2 |
| 0.007 | 0.014 | 0.628 | 31.3 |
| 0.029 | 0.016 | 0.075 | 41.2 |
| -0.024 | 0.014 | 0.078 | 30.1 |
| -0.007 | 0.014 | 0.593 | 96.0 |
| 0.004 | 0.014 | 0.745 | 37.1 |
| 0.000 | 0.014 | 0.998 | 44.7 |
| 0.019 | 0.014 | 0.172 | 33.9 |
| -0.001 | 0.014 | 0.962 | 30.0 |
| 0.013 | 0.017 | 0.465 | 41.7 |
| -0.004 | 0.015 | 0.805 | 33.0 |
| -0.027 | 0.014 | 0.049 | 30.7 |
| 0.026 | 0.018 | 0.158 | 38.0 |
| 0.006 | 0.014 | 0.647 | 31.1 |
| 0.026 | 0.014 | 0.054 | 45.5 |
| 0.002 | 0.014 | 0.879 | 38.5 |
| -0.009 | 0.014 | 0.517 | 30.7 |
| 0.005 | 0.020 | 0.802 | 30.1 |
| 0.028 | 0.014 | 0.040 | 44.4 |
| -0.025 | 0.014 | 0.080 | 41.6 |
| 0.010 | 0.016 | 0.545 | 30.6 |
| 0.003 | 0.014 | 0.850 | 31.9 |
| -0.009 | 0.015 | 0.535 | 32.1 |
| 0.024 | 0.017 | 0.152 | 37.4 |
| 0.001 | 0.014 | 0.925 | 30.6 |
| -0.008 | 0.014 | 0.578 | 31.9 |
| -0.001 | 0.016 | 0.966 | 38.8 |
| 0.013 | 0.017 | 0.451 | 31.7 |
| -0.020 | 0.014 | 0.159 | 32.6 |
| -0.036 | 0.015 | 0.014 | 55.6 |
| 0.002 | 0.017 | 0.924 | 32.9 |
| 0.032 | 0.014 | 0.027 | 29.8 |
| -0.017 | 0.014 | 0.237 | 58.0 |
| -0.002 | 0.016 | 0.889 | 32.1 |
| 0.022 | 0.014 | 0.123 | 37.7 |
| -0.024 | 0.017 | 0.163 | 36.7 |
| -0.004 | 0.014 | 0.784 | 32.5 |
| 0.005 | 0.016 | 0.726 | 41.0 |
| 0.022 | 0.014 | 0.114 | 46.8 |

Abbreviations: GERD, gastroesophageal reflux disease; SNP, single nucleotide polymorphism; pos, position; chr, chromosome; SE, standard error; MR, Mendelian randomization.
