# Supplementary material for: Causal associations of gastroesophageal reflux disease with sepsis and sepsis mortality within 28 days: A bidirectional 2-sample Mendelian randomization study
Source: Medicine (Baltimore). 2026 Sep 4;105(36):e50553. doi: 10.1097/MD.0000000000050553 (PMC13549649; doi:10.1097/MD.0000000000050553)
Supplement: Supplementary file 3 [file medi-105-e50553-s003.docx]

Table S5. The causal effects of exposures on outcomes.

| exposure | outcome | nsnp | method | b | se | pval |
| --- | --- | --- | --- | --- | --- | --- |
| GERD | sepsis | 62 | MR Egger | 0.215 | 0.383 | 0.577 |
|  |  |  | Inverse variance weighted | 0.306 | 0.058 | 1E-07 |
|  |  |  | Weighted median | 0.254 | 0.083 | 0.002 |
|  |  |  | Weighted mode | 0.083 | 0.203 | 0.683 |
| GERD | sepsis (28-day death) | 62 | MR Egger | 1.442 | 0.972 | 0.143 |
|  |  |  | Inverse variance weighted | 0.365 | 0.148 | 0.014 |
|  |  |  | Weighted median | 0.403 | 0.208 | 0.053 |
|  |  |  | Weighted mode | 0.952 | 0.616 | 0.127 |
| sepsis | GERD | 4 | MR Egger | 0.587 | 0.284 | 0.175 |
|  |  |  | Inverse variance weighted | 0.025 | 0.048 | 0.602 |
|  |  |  | Weighted median | 0.030 | 0.044 | 0.494 |
|  |  |  | Weighted mode | 0.092 | 0.085 | 0.357 |
| sepsis (28-day death) | GERD | 5 | MR Egger | 0.045 | 0.102 | 0.689 |
|  |  |  | Inverse variance weighted | 0.034 | 0.014 | 0.015 |
|  |  |  | Weighted median | 0.039 | 0.019 | 0.044 |
|  |  |  | Weighted mode | 0.040 | 0.024 | 0.167 |

| or | or_lci95 | or_uci95 |
| --- | --- | --- |
| 1.240 | 0.585 | 2.629 |
| 1.358 | 1.212 | 1.522 |
| 1.289 | 1.096 | 1.516 |
| 1.087 | 0.731 | 1.616 |
| 4.229 | 0.629 | 28.418 |
| 1.440 | 1.078 | 1.924 |
| 1.497 | 0.995 | 2.251 |
| 2.590 | 0.775 | 8.662 |
| 1.799 | 1.031 | 3.139 |
| 1.025 | 0.934 | 1.126 |
| 1.031 | 0.945 | 1.124 |
| 1.096 | 0.929 | 1.294 |
| 1.046 | 0.857 | 1.277 |
| 1.035 | 1.007 | 1.063 |
| 1.039 | 1.001 | 1.079 |
| 1.041 | 0.993 | 1.090 |

Abbreviations: GERD, gastroesophageal reflux disease; nsnp, number of single nucleotide polymorphism; OR, odd ratio; CI, Confidence interval; SE, standard error.
