# Supplementary material for: Causal associations of gastroesophageal reflux disease with sepsis and sepsis mortality within 28 days: A bidirectional 2-sample Mendelian randomization study
Source: Medicine (Baltimore). 2026 Sep 4;105(36):e50553. doi: 10.1097/MD.0000000000050553 (PMC13549649; doi:10.1097/MD.0000000000050553)
Supplement: Supplementary file 4 [file medi-105-e50553-s006.docx]

Table S3. Genetic data for the MR analysis on the effect of sepsis on GERD.

| SNP | chr.exposure | pos.exposure | effect allele | reference allele | exposure | | |
| --- | --- | --- | --- | --- | --- | --- | --- |
|  |  |  |  |  | beta | se | p |
| rs11068069 | 12 | 1E+08 | T | C | 0.083 | 0.018 | 3.60E-06 |
| rs11980516 | 7 | 4E+07 | T | G | 0.089 | 0.018 | 9.50E-07 |
| rs2226602 | 11 | 9E+07 | T | A | 0.076 | 0.016 | 3.00E-06 |
| rs3851566 | 10 | 1E+08 | G | A | 0.063 | 0.014 | 4.63E-06 |

| outcome | | | F statistics |
| --- | --- | --- | --- |
| beta | se | p |  |
| 0.009 | 0.006 | 0.147 | 21.5 |
| 0.009 | 0.006 | 0.170 | 24.0 |
| -0.003 | 0.006 | 0.598 | 21.8 |
| -0.005 | 0.005 | 0.297 | 21.0 |

Abbreviations: GERD, gastroesophageal reflux disease; SNP, single nucleotide polymorphism; pos, position; chr, chromosome; SE, standard error; MR, Mendelian randomization.
