# Supplementary material for: Causal associations of gastroesophageal reflux disease with sepsis and sepsis mortality within 28 days: A bidirectional 2-sample Mendelian randomization study
Source: Medicine (Baltimore). 2026 Sep 4;105(36):e50553. doi: 10.1097/MD.0000000000050553 (PMC13549649; doi:10.1097/MD.0000000000050553)
Supplement: Supplementary file 5 [file medi-105-e50553-s007.docx]

Table S4. Genetic data for the MR analysis on the effect of sepsis mortality within 28 days on GERD.

| SNP | chr.exposure | pos.exposure | effect allele | reference allele | exposure | | |
| --- | --- | --- | --- | --- | --- | --- | --- |
|  |  |  |  |  | beta | se | p |
| rs17090050 | 18 | 5E+07 | A | G | -0.175 | 0.038 | 4.86E-06 |
| rs177999 | 18 | 2E+07 | T | C | 0.201 | 0.044 | 4.39E-06 |
| rs7155416 | 14 | 8E+07 | T | G | 0.243 | 0.053 | 4.38E-06 |
| rs824453 | 2 | 2E+07 | C | T | 0.223 | 0.047 | 1.85E-06 |
| rs9955473 | 18 | 7E+07 | C | T | -0.169 | 0.036 | 3.02E-06 |

| outcome | | | F statistics |
| --- | --- | --- | --- |
| beta | se | p |  |
| -0.002 | 0.006 | 0.768 | 20.9 |
| 0.008 | 0.006 | 0.195 | 21.1 |
| 0.002 | 0.008 | 0.769 | 21.1 |
| 0.015 | 0.007 | 0.031 | 22.7 |
| -0.007 | 0.005 | 0.170 | 21.8 |

Abbreviations: GERD, gastroesophageal reflux disease; SNP, single nucleotide polymorphism; pos, position; chr, chromosome; SE, standard error; MR, Mendelian randomization.
